# Development of a Novel Mule Deer Genomic Assembly and Species-Diagnostic SNP Panel for Assessing Introgression in Mule Deer, White-Tailed Deer, and Their Interspecific Hybrids

**DOI:** 10.1534/g3.118.200838

**Published:** 2019-01-22

**Authors:** Ty Russell, Catherine Cullingham, Arun Kommadath, Paul Stothard, Allen Herbst, David Coltman

**Affiliations:** * Department of Biological Sciences, University of Alberta, Edmonton, Alberta, Canada, T6G 2E9; † Department of Agricultural Food and Nutritional Science, University of Alberta, Edmonton, Alberta, Canada, T6G 2E9

**Keywords:** mule deer, white-tailed deer, hybridization, introgression, SNP, genome

## Abstract

Mule deer (*Odocoileus hemionus*) are endemic to a wide variety of habitats in western North America, many of which are shared in sympatry with their closely related sister-species white-tailed deer (*Odocoileus virginianus*), whom they hybridize with in wild populations. Although mule deer meet many ideal conditions for a molecular ecological research species, such as high abundance, ecological importance, and broad dispersal and gene flow, conservation genetic studies have been limited by a relative lack of existing genomic resources and inherent difficulties caused by introgression with white-tailed deer. Many molecular tools currently available for the study of cervids were designed using reference assemblies of divergent model species, specifically cattle (*Bos taurus*). Bovidae and Cervidae diverged approximately 28 million years ago, therefore, we sought to ameliorate the available resources by contributing the first mule deer whole genome sequence draft assembly with an average genome-wide read depth of 25X, using the white-tailed genome assembly (Ovir.te_1.0) as a reference. Comparing the two assemblies, we identified ∼33 million single nucleotide polymorphisms (SNPs) and insertion/deletion variants. We then verified fixed SNP differences between the two species and developed a 40-loci SNP assay capable of identifying pure mule deer, white-tailed deer, and interspecific hybrids. Assignment capacity of the panel, which was tested on simulated datasets, is reliable up to and including the third backcross hybrid generation. Identification of post-F1 hybrids will be necessary for hybrid zone population studies going forward, and the new mule deer assembly will be a valuable resource for genetic and comparative genomics studies.

Hybridization is not uncommon among plant and animal taxa worldwide (Schwenk *et al.* 2008). Mallet (2005) estimated that 10–30% of multicellular plant and animal species hybridize regularly and that, of those species, between 1 in 100 and 1 in 10 000 sympatric individuals are hybrids. Introgression has been identified as a mechanism for generating progressive evolutionary events such as novelty, divergent selection, and speciation (Dowling and Secor 1997; Lamer *et al.* 2015; Seehausen 2004) though it can also complicate management and conservation by compromising coadapted gene complexes (Edmands *et al.* 2009), morphological discernment (Leary *et al.* 1996), local adaptation (Martinsen *et al.* 2001), and the genetic integrity of unique phylogenetic lineages (Rhymer and Simberloff 1996). For these reasons, genetic tools to monitor current distributions and degrees of hybridization will be valuable for future researchers, policy-makers, hunters, and conservationists.

Introgressive hybridization tends to occur most frequently among sympatric, closely related species in rapidly diversifying adaptive radiations (Abbott *et al.* 2013; Gourbière and Mallet 2010; Price and Bouvier 2002) such as between mule deer (MD) (*Odocoileus hemionus*) and its sister species, white-tailed deer (WT) (*O. virginianus*), in the prairies of Western North America (Bradley *et al.* 2003 ; Cronin 1991; Hornbeck and Mahoney 2000; Stelfox and Adamczewski1993). Cervids constitute cornerstone taxa in ecological, economic, and cultural sectors of Western Canada. The output value of elk and deer farms, before indirect spillover effects into other industries, is estimated to be more than $43 million in Canada with Alberta being responsible for over a quarter of that total (Petigara *et al.* 2011). As well, total big game hunting expenditures by Canadian residents in 2012 exceeded $1 billion with $169 million coming from Albertans (Federal, Provincial, and Territorial Governments of Canada 2014). As of 2018, Canadian federal and provincial laws do not recognize hybrid deer as a separate entity from the parental species, which can create confusion as to hunting and harvesting regulations. Access to tools capable of identifying hybrid individuals and characterizing the rate of hybridization in wild populations could facilitate the implementation of management standards. Hybridization may also play a role in the spread of chronic wasting disease (CWD), a transmissible spongiform encephalopathy (TSE) that affects both focal *Odocoileus spp*. (Miller *et al.* 2012; Williams 2005). CWD has been reported to be more prevalent in MD relative to WT in areas of sympatry (Habib *et al.* 2011; Miller *et al.* 2000). Behavioral differences between species, in part, account for this asymmetry (Cullingham *et al.* 2010; Cullingham *et al.* 2011) but genetic polymorphisms have also been implicated (Wilson *et al.* 2009). Considering that hybrids potentially provide an opportunity to bridge disease transmission across species, further research of CWD susceptibility and pervasiveness among hybrids may provide insight into the transmission dynamics.

Identification of hybrid deer using morphological traits alone is rarely done with confidence. Coloration and antler shape are not always intermediate between parental phenotypes. The most consistent and accessible morphological marker appears to be the metatarsal gland, which is intermediate in both position and size (Carr *et al.* 1986; Wishart 1980). The biomechanics of the escape gait may serve as another indicator. While the stot of the MD is highly differentiated from the gallop of the WT, the bound of the hybrid is highly variable, even between strides of the same individual, and seems to be wholly inefficient (Lingle 1993). Molecular markers used to identify hybrids include serum albumin electrophoresis (Hornbeck and Mahoney 2000), a ribosomal 28S DNA marker (Bradley *et al.* 2003), and mitochondrial endonuclease recognition site mapping (Carr *et al.* 1986). None of these methods, however, are reliably informative of backcrosses after the F1 generation. Single nucleotide polymorphisms (SNPs) have proven effective in investigations of admixture between divergent taxa in several hybrid systems (Stephens *et al.* 2009; Twyford and Ennos 2011; Wiley *et al.* 2009; Cullingham *et al*. 2013; Lamer *et al*. 2014). This is due, in part, to their high abundance in the genome which improves discriminating power and facilitates the recognition of varying levels of introgression. Furthermore, SNPs are more consistently reproducible, easier to automate, and more stable in mammals than microsatellites (Fernández *et al.* 2013; Tokarska *et al.* 2009; Vignal *et al*. 2002). An added benefit of SNPs is that their biallelic nature is convenient for the discernment of two different species.

A species-discriminating SNP assay will effectively describe two traits of hybridization: the classification of hybrids based on the presence of species-specific alleles and a measure of hybridity based on the proportions of those alleles. Quantification of the varying introgression depths of hybridized populations will provide a snapshot with more resolution than would be available from observation of the F1 individuals alone. Detection power of this magnitude is necessary to explore hybrid zone structure because current hypotheses predict reduced fitness in F1 individuals (Hornbeck and Mahoney 2000); therefore, the frequency and abundance of F1’s in the wild is not a suitable proxy for the occurrence of advanced-generation backcrosses. The ability to resolve backcross generation status will be informative of hybrid productivity and directionality. By differentiating advanced-generation backcrosses from pure breeding individuals, the rate of false negative hybrid diagnoses will drastically decrease, effectively increasing hybrid sample size and lineage diversity as well as our understanding of hybridization frequency and geographical distribution (Lamer *et al.* 2015). The identification of the degree of introgression is crucial for determining the structure and stability of the population and the entire hybrid zone in which it resides. For example, whether selection is working for or against hybrids can help to elucidate the type of suture zone present and, from that information, we can draw inferences and predictions about the hybrid zone itself (Hu 2005). By better understanding the structure of the populations that make up the hybrid zone, we improve our ability to forecast its future.

Currently, molecular resources suitable for application in *Odocoileus spp*. exist as sets of various individual loci but none capture mass sequence data capable of providing a reference to which polymorphisms can be mapped. Along with assorted microsatellite loci (Bishop *et al.* 1994; DeWoody *et al.* 1995; Jones *et al.* 2000; Wilson *et al.* 1997), current SNP datasets have been engineered by subjecting MD, black-tailed deer (*O. h. columbianus*), and WT to genotyping on the BovineSNP50 Bead Chip (Haynes and Latch 2012); bovine exon-targeted resequencing (Powell *et al.* 2016); and mapping high-throughput next-generation sequencing data to an existing bovine reference genome assembly (Brauning *et al.* 2015). All of these datasets, however, relied heavily upon previously available bovine genomic resources for mapping and/or template purposes, thereby losing data in regions not conserved after the divergence of Cervidae and Bovidae some 28 million years ago (Hassanin *et al.* 2012). A whole genome sequence (WGS) assembly of MD will function as a more suitable reference for future polymorphism mining in Cervids and increase accessibility to regions of the genome not conserved from Bovidae.

In this study, we present two novel genomic resources targeted for the study of MD and MD X WT hybrids: a draft WGS assembly of the MD and a species-diagnostic SNP panel. The MD WGS data are a versatile and informative dataset for cervid researchers investigating the genetic basis of traits, disease susceptibilities, and variants by providing a reference genome to which polymorphisms can be mapped (Ng and Kirkness 2010). The species-diagnostic SNP panel provides a method to reliably differentiate between MD and WT as well as to identify hybrids and quantify introgressive geneflow, even in the limited amounts present in advanced-generation backcrosses.

## Materials and methods

### Reference-guided MD Genome Assembly and Annotation

Total DNA was isolated from the ear tissue of a hunter harvested MD. The ear was shaved to remove hair and subsequently powdered by grinding under liquid nitrogen with a mortar and pestle. DNA was extracted from the powdered ear tissue (0.1g) for one hour at 68 degrees centigrade with 500µl of an extraction buffer containing 2% cetrimonium bromide, 100mM TrisCl pH 7.5, 1.4M NaCl, 20mM EDTA, 2% polyvinylpyrrolidine, 0.2% mercaptoethanol, 100µg/mL proteinase K, and 20µg/mL RNase A. An equal volume of chloroform/isoamyl alcohol (24:1) was added, the sample centrifuged for 5 min at 14,000 × g and the aqueous phase collected. The aqueous phase was extracted with tris saturated phenol/chloroform, centrifuged and again the aqueous phase collected. Finally, the DNA was precipitated with 2.5 volumes of 95% ethanol and 0.1 volume of sodium acetate. The pellet was washed with 70% ethanol, briefly dried to remove residual ethanol, and dissolved in water. DNA quality was assessed by UV-Vis spectroscopy and agarose gel electrophoresis.

Genomic DNA was sequenced on an Illumina HiSeq X Ten sequencer (shotgun PCR free library; paired-end sequencing; 150 bp reads). Read quality was assessed using FastQC software (available at: http://www.bioinformatics.babraham.ac.uk/projects/fastqc/) prior to and after performing quality control (QC) steps. The QC included quality based read trimming and adapter removal using Trimmomatic (Bolger *et al.* 2014) followed by adapter and phiX read removal using Cutadapt (Martin 2011) with default settings for both software. Reads that passed QC were mapped to the WT deer reference genome assembly (Ovir.te_1.0; GenBank assembly accession: GCA_002102435.1) using the BWA-MEM algorithm of the Burrows-Wheeler Alignment tool (Li 2013) with default settings. Mapped reads were sorted and indexed using SAMtools (Li *et al.* 2009). Duplicate reads were tagged using MarkDuplicates software from Picard tools (available at: http://broadinstitute.github.io/picard). Variant calling and MD consensus sequence generation were performed using a combination of mpileup, bcftools and vcfutils.pl scripts from SAMtools. Identified SNPs were annotated using SnpEff (Cingolani *et al.* 2012). Regions with no coverage on the consensus sequence were filled with N’s.

### Selection and Validation of Species-discriminating SNPs

DNA was extracted from a sampling group of multiple species of deer as part of another study whose purpose was to aggregate and identify markers that would be useful to the New Zealand deer industry for parentage assignment and sub-species differentiation (Rowe *et al.* 2015). Samples were genotyped on Illumina 50K CervusSNP50, 24-sample Bead Chips (Illumina, San Diego), a high-throughput SNP assay developed for deer (*Cervus* genus) by comparing genomic sequence data from red deer, wapiti (subspecies of *Cervus elaphus*), and sika (*Cervus nippon*) by Brauning *et al.* (2015). A set of 44,448 SNPs were genotyped in 396 individuals, including 17 WT and 8 MD from Alberta and Saskatchewan, Canada. Loci and samples exhibiting non-autosomal and non-Mendelian inheritance patterns, low call rates, and duplication were excluded (Rowe *et al.* 2015). The GenABEL package in R software (Aulchenko *et al.* 2007) was used to carry out genotype quality control measures. We identified 129 loci that had call rates >0.7 in both WT and MD and also appeared to show fixed differences between the two species, *i.e.*, biallelic loci where one allele is homozygous in WT and the other in MD.

A preliminary validation step was implemented by aligning the 201 bp sequences that include the SNP and its 100 bp flanking sequences to the new MD genome assembly generated here and to the WT genome assembly (Ovir.te_1.0). This allowed us to eliminate 10 SNPs that were non-discriminating or otherwise unable to be mapped prior to assay design and further validation, leaving 119 species-discriminating loci, from which a subset of 40 were randomly selected for validation. Because the morphology of hybrids varies and their frequency in wild populations as of now can only be approximated, the genetic purity of deer from Alberta should not be assumed. Even minute levels of introgression in individuals believed to be pure could compromise the efficacy of species delimitation. As a precaution, we minimized the risk of ancestral hybridization by importing samples from regions of allopatry, where hybridization is less common or non-existent. MD are native to western North America; populations are rarely found further east than western Minnesota or Iowa (Patterson *et al.* 2003). For this reason, we used WT samples collected in Ontario (n = 10). WT distribution spans continental North America but excludes most of Utah and Nevada (Hewitt 2011). MD samples used for validation were collected from Utah (n = 2), Nevada (n = 5), and Montana (n = 3). Note that Montana is a known region of sympatry and thus should be considered as quantitative support of the other two MD sampling locations but would otherwise not be used independently. As well, samples of *a priori* hybrid heredity (n = 10) were obtained from Alberta Environment and Parks. Pedigree records were available for these individuals, as they were intentionally bred as hybrids as part of a long-term study in the mid-1980s (Wishart *et al.* 1988). DNA was extracted using a Qiagen 96 DNeasy Blood and Tissue kit following the manufacturer’s instructions (Qiagen, Mississauga, Ontario, Canada) and genotyped on the final panel. Genotypes of these individuals are listed in supplementary table S1.

### SNP Assay Assignment Efficacy

*HybridLab* software (Nielsen *et al.* 2006) uses classical genetics principles to simulate offspring genotypes from a set of observed parental genotypes. Two populations of pure and hybrid genotypes were independently simulated in *HybridLab* using the parental genotypes of 10 pure MD from Utah, Nevada, and Montana and 10 pure WT from Ontario. Simulated populations consisted of 100 individuals in each of 10 hybrid generation classes: parental WT, parental MD, F1, F2, and three backcross generations of each species. Posterior probabilities of assignment for simulated populations were computed by *NewHybrids* version 1.1 (Anderson and Thompson 2002) using 500,000 burnin iterations preceding 500,000 sweeps. Genotype frequency classes were set in *NewHybrids* using Jeffrey’s-like priors (Table 1). Repetitions were also done using two and four backcross generations (data not shown): fourth backcrosses could not be distinguished from third backcrosses or pure-species and, upon retraction of the third backcross as an assignment option, the post-secondary backcross individuals were erroneously assigned to the second backcross generation. For these reasons, the third backcross generation was deemed as the most advanced introgression level detectable. *NewHybrids* assignments were tested for efficacy using the R package *hybriddetective* (Wringe *et al.* 2017). *hybriddetective* is designed to analyze hybrid assignments as deep as one backcross generation, therefore, some code was amended to account for two additional levels of introgression (available upon request). Assignment probabilities for simulated populations, as calculated by *NewHybrids*, were evaluated by *hybriddetective* using three metrics: accuracy, the proportion of assignments to a particular category that were correct ([correct assignments] / [total assignments of a particular category]); efficiency, the proportion of individuals in a particular category that were assigned correctly ([correct assignments] / [total individuals of a particular category]); and power, the product of accuracy and efficiency (Vähä and Primmer 2006).

**Table 1:** Assignment criteria and category definition of white-tailed (WT) and mule deer (MD) hybrids. Heterozygous and homozygous genotype frequencies are required as input prarmeters in NewHybrids to specify the number and configuration of hybrid classes

		Genotype frequency			
NewHybrid category		WT (AA)	H (AB)	MD (BB)	NewHybrid category expanded
WT	Parental WT	1	0	0	WT
MD	Parental MD	0	0	1	MD
F1	First-generation hybrid	0	1	0	(WT x MD)
F2	Second-generation hybrid	0.25	0.5	0.25	((WT x MD) x (WT x MD))
BxWT	First-generation backcross	0.5	0.5	0	(WT x (WT x MD))
BxMD	First-generation backcross	0	0.5	0.5	(MD x MD x WT))
Bx2WT	Second-generation backcross	0.75	0.25	0	(WT x (WT x (WT x MD)))
Bx2MD	Second-generation backcross	0	0.25	0.75	(MD x (MD x (MD x WT)))
Bx3WT	Third-generation backcross	0.875	0.125	0	(WT x (WT x (WT x (WT x MD))))
Bx3MD	Third-generation backcross	0	0.125	0.875	(MD x (MD x (MD x (MD x WT))))

### Data Availability

See Supplementary Table 1 for species-discriminating loci names and genotypes of individuals used for validation. All SNP loci are available from Brauning *et al.* (2015). This Whole Genome Shotgun project has been deposited at DDBJ/ENA/GenBank under the accession RCHL00000000. The version described in this paper is version RCHL01000000. Supplemental material available at Figshare: https://doi.org/10.25387/g3.7250309.

## Results

### MD Genome Assembly, Variant Identification and Annotation

A total of 378,654,417 paired-end reads were obtained in fastq format files. Following quality control steps, 334,409,387 paired-end reads remained that were then aligned to the WT reference genome assembly. A total of 536,357,624 reads were mapped (mapping rate of 75%) of which 81.69% were properly paired. The average genome-wide coverage and GC content of mapped reads were 25X and 40% respectively. Variant calling on mapped reads compared to the WT reference resulted in approximately 30 million SNPs and 2.9 million INDELs. After filtering at SNP mapping quality Q20, the variants ranged from 30.1M at read depth 10 to 32.9M at read depth 2. The genome-wide ratio of transitions to transversions at read depth 5 and Q20 was 2.4, which is similar to the value reported in human studies (DePristo *et al.* 2011; Jun *et al.* 2015).

### Species-discriminating SNP Assay

**Figure 1:**
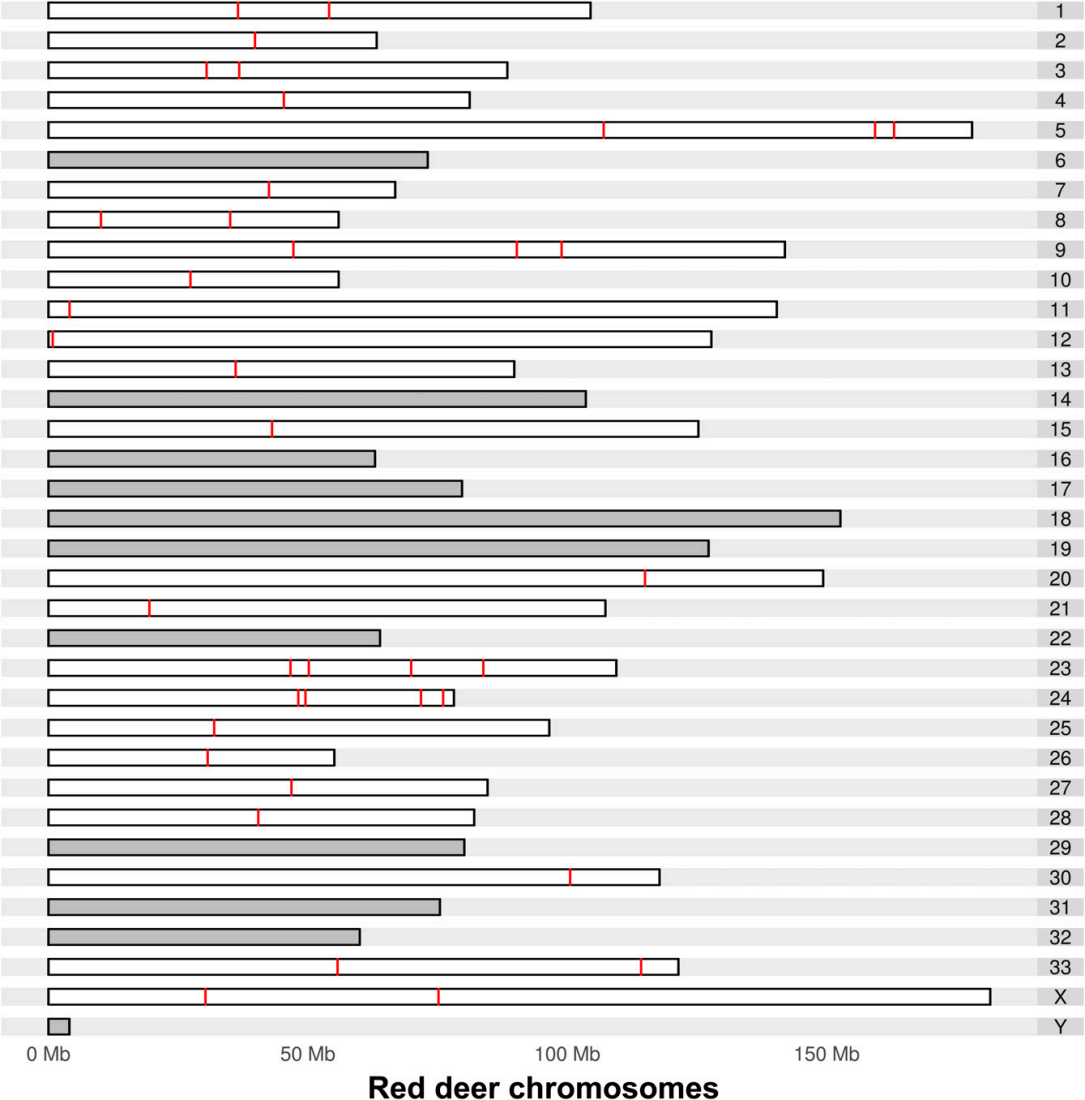
MD/WT species discriminating SNP loci mapped to Red Deer genome assembly. Red bars indicate the positions on the Red deer chromosomes that the 40 loci SNP assay mapped to. The mapping positions were determined by alignments of the SNP loci along with the 100 bp flanking sequences onto the Red deer genome assembly (CerEla1.0) using BLAT. The loci covered 24 of the 35 chromosomes, with some chromosomes harboring multiple loci (chromosomes not covered are shaded gray). One of the loci mapped to an unplaced contig (not depicted here).

**Figure 2:**
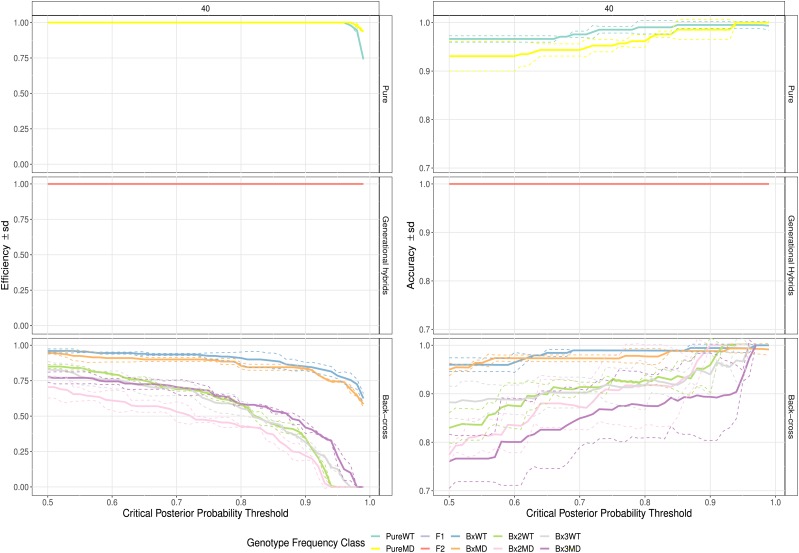
Hybrid assignment efficacy as determined by the hybridPowerComp function in the R package hybriddetective. Facets labeled as Pure indicate parental WT and MD, Generational Hybrids refers to F1 and F2 individuals (lines overlaid), and Back-cross includes first, second, and third generation backcrosses of both species. Accuracy was calculated as [correct assignments] / [total assignments of a particular category]. Efficiency was calculated as [correct assignments] / [total individuals of a particular category].

Based on aligning the selected loci and their 100 bp flanking sequences to the genome assembly of the Red Deer (CerEla1.0, GenBank: GCA_002197005.1), which is finished at chromosomal level, we determined that the 40 loci were distributed across 24 of the 35 Red deer chromosomes (Figure 1), indicating good genome-wide distribution. SNP genotypes of 30 individuals, including pure MD, pure WT, and *a priori* hybrids, were successfully called at all 40 loci. Because all loci were highly discriminating, pure-breeding, F1, and F2 individuals were assigned with predictable confidence: all reached 100% efficiency and greater than 93% accuracy at a critical posterior probability threshold of 0.50. Likewise, first-generation backcrosses were detected at ∼95% efficiency and accuracy at the same threshold. Second- and third-generation WT backcrosses were assigned with slightly more proficiency than their MD counterparts; advanced-generation WT backcross assignments were accurate and efficient at a rate of ∼85%, while the same MD assignments achieved rates of ∼75%. Accuracy increased with the probability threshold, but efficiency and sample size decreased (Figure 2). Both of these trends agree with expected outcomes. The critical probability threshold will be subjective based on the user’s research question; by increasing the threshold, some samples assigned with a lower probability will be excluded but those that remain are more likely to be assigned correctly. For all samples to be assigned to a hybrid category, a probability threshold of 0.5 should be used. The power of assignment for a specific hybrid category slowly declined as the probability threshold was increased from 0.5 to 0.8, then decreased more drastically. Alternatively, the power of assignment for hybrids in general (*i.e.*, all hybrid categories combined) remained relatively stable over a wider range of probability thresholds: 0.80 at a critical probability threshold of 0.5 and 0.69 at a threshold of 0.9.

## Discussion

The development of the MD genome assembly helped us to identify fixed differences between WT and MD, and to develop a 40-loci SNP assay capable of reliably classifying pure WT, pure MD, and their interspecific hybrids into introgression categories. This diagnostic panel overcomes several limitations encountered by previous methods used in deer hybridization analyses, including: lack of assignment power due to low marker abundance, ambiguity of distinguishing morphological traits, inconsistent reproducibility, and inability to resolve advanced-generation backcrosses. Along with a solution to these deficiencies, the whole genome sequence, to which these and other polymorphisms can be mapped, improves accessibility to regions of the MD genome previously withheld from analyses by divergence from model research species. Together, these resources bear valuable conservation potential for researchers, hunters, farmers, and environmentalists.

Hybrid populations simulated in duplicate using genotypes of pure-species from regions of allopatry demonstrated that the SNP assay diagnostic strength is reliable up to and including the third backcross generation. Simulations were composed of 10 hybrid classes (n = 100 for each class), including both pure species, F1 and F2 hybrids, and 3 backcross generations of each species (Table 1). We chose not to include simulations for individuals with pedigrees containing multiple hybrids (*e.g.*, backcross X backcross or backcross X F1/F2 scenarios) because the differences in individual genotype frequencies are very low between respective classes and any attempt to distinguish those classes with medium-throughput genotype data would be impractical. Furthermore, Wishart *et al.* (1988) found male F1 hybrids to be almost exclusively sterile, significantly decreasing the likelihood of encountering, in a wild population, an F2 or other product of hybrid X hybrid pairings. This apparent adherence to Haldane’s rule (Haldane 1922), which predicts infertility in the heterogametic sex of interspecific hybrids, is just one opportunity for future research in a complex hybrid system that now has the molecular resources for further investigation. Another question of interest is the directionality of hybridization. In the same study, more viable offspring were produced from crosses under controlled laboratory conditions between MD males and WT females than in the reciprocal cross (Wishart *et al.* 1988); however, studies of hybrids in wild populations have both supported this finding (Carr *et al.* 1986) and refuted it (Carr and Hughes 1993; Cathey *et al.* 1998; Stelfox and Adamczewski 1993). Behavioral traits such as boldness and promiscuity likely play major roles in this dynamic (Lingle *et al.* 2007a; Lingle, *et al.* 2007b) but genetic incompatibilities may also contribute, as indicated by increased spontaneous abortions of fetuses generated by MD female X WT male matings (Wishart *et al.* 1988). This assay will be instrumental in validating observations such as these with empirical data from the field by alleviating reliance on pedigree records. By confirming or disproving the persistence of these trends in wild populations, we can explore the structural dynamics of the hybrid zone with more resolution than has been available in the past. Furthermore, the focal hybrid system is not confined to western Canada; studies have reported MD x WT hybridization in West Texas and Montana (Carr *et al.* 1986; Cronin 1991). Whether these zones are consistent or clinal in hybridization frequency is yet to be investigated. Rusek *et al.* (2015) noted that, although hybrid complexes are not uncommon in nature, their evolutionary inception and subsequent persistence remains relatively ambiguous and that studies targeted toward fine-scale genetic analyses of such systems are largely limited by lack of resources capable of identifying and quantifying introgression.

Previous sets of molecular markers and a few diagnostic morphological traits were successful at identifying F1 hybrids with some degree of accuracy but none had the diagnostic power to differentiate subsequent backcross generations; many backcross hybrids were mistaken either as F1’s or as pure-species. Without the ability to interrogate more advanced generations, the underlying factors balancing the co-existence of hybrids with parental species has remained inaccessible to researchers and conservationists (Hornbeck and Mahoney 2000). Such factors are both ecological and genetic in nature and typically include reproductive barrier strength, vigor and fertility of hybrids, pathogen pressure, and selection by predation (Daum *et al.* 2012; Duenez-Guzman *et al.* 2009; Hall *et al.* 2006; Wolf *et al.* 2001). The first step to elucidating the specific conditions of these factors as they pertain to the focal system is to delve deeper into the structure and distribution of hybridized populations via a large-scale study. With access to advanced-generation backcrosses, we now have the detection power necessary to capture a robust snapshot of the hybrid landscape. Population data will be imperative to resolve broad questions about the hybrid zone in which the *Odocoileus* system resides. For example, which model does it most resemble? Endogenous selection against hybrids (heterozygote breakdown) is indicative of a tension zone (Hewitt 1988), whereas exogenous selective pressure potentially driven by niche divergence in the parental species is more consistent with the geographical selection-gradient model (Johnson *et al.* 2016). A third possibility would be that hybrids fill some ecological niche and are being selected for. Until a population-scale study is undertaken, we can only theorize. The results of that study will not only advance the collective body of knowledge of hybrid zones, but more generally, that of cervid evolution and ecology in western North America at a time when they are faced with the looming threat of chronic wasting disease. CWD is a fatal neurodegenerative disorder that has proven highly transmissible in wild populations (Williams *et al.* 2002). At the time of this writing, CWD is endemic in two Canadian provinces, Alberta and Saskatchewan, and at least 23 states in the continental United States, including some near the Canadian border such as Minnesota, Wisconsin, Michigan, Pennsylvania, New York, and both Dakotas (Haley and Hoover 2015; Richards 2018; see also https://www.cdc.gov/prions/cwd/occurrence.html). Containing the spread of CWD continues to be a high priority for governmental bodies, researchers, and conservationists because of the cultural, economic, and ecological importance of cervids in Canada and the United States.

The MD whole genome sequence presented here may also serve to progress the study of CWD. Genetic polymorphisms of the *Prnp* gene, which encodes the protein that is eventually converted into the infectious agent, have been associated with varied disease incubation periods (Wilson *et al.* 2009). Our assembly provides an ideal reference to which these and other variable sites can be mapped, while also serving as a localizing template for structures of interest, such as primer design. The utility of this functional, all-purpose *Odocoileus* reference lies in alleviating the reliance on model-species genomes that may be less recently diverged. In doing so, ease of access to novel regions of the genome, including those not conserved in Bovidae and undocumented subtleties such as promoters, splice sites, and introns, will be vastly improved. Facilitating access to these untapped domains will effectively foster the development of more specific molecular advances applicable to a range of research questions throughout the *Odocoileus* genera.
